# Supplementary material for: Evaluation of a game-based training course to build capacity for insecticide resistance management in vector control programmes
Source: PLoS One. 2020 Oct 15;15(10):e0240514. doi: 10.1371/journal.pone.0240514 (PMC7561117; doi:10.1371/journal.pone.0240514)
Supplement: S1 Appendix — (PDF) [file pone.0240514.s001.pdf]

1) What type of data does human landing catch give you, but light traps do not?

   a. Seasonal fluctuations in mosquito density
   b. Indoor resting density
   c. Biting times
   d. Estimate of host-seeking mosquito density

2) Which collection method is most appropriate for accurately estimating the entomological inoculation rate (EIR)?

   a. Light trap catch
   b. Pyrethrum spray catch
   c. Larval collections
   d. Human landing catch

3) The most important criterion in selecting where to collect mosquitoes for a routine entomological monitoring programme is:

   a. Ease of access
   b. Abundance of mosquitoes
   c. Whether regular collections have been done there previously
   d. Species composition

4) To obtain adult insecticide susceptibility data, wild mosquito populations are collected and taken back to the insectary, where…

   a. Mosquitoes are transferred to a clean, closed container, and are sprayed with a known dose of insecticide. The number of mosquitoes that die over a certain amount of time is recorded and compared to a particular threshold.
   b. A surface is treated with a known dose of insecticide. The mosquitoes are exposed to the surface by placing them in a plastic cone attached to the surface. The number of mosquitoes that die over a certain amount of time is recorded and compared to a particular threshold.
   c. The inside of a container is treated with a known dose of insecticide. The mosquitoes are exposed to the insecticide by putting them inside the container. The number of mosquitoes that die after a certain amount of time is recorded and compared to a particular threshold.
   d. A piece of paper that has been treated with a known dose of insecticide is placed in the cage with the mosquitoes. The number of mosquitoes that die after a certain amount of time is recorded and compared to a particular threshold.

5) Which of the following methods can you use to determine the mechanism of resistance?

   a. Synergist bioassays and PCR
   b. PCR and cone bioassays
   c. WHO tube bioassays and synergist bioassays
   d. WHO tube bioassays and PCR

6) The resistance profile of a population of mosquitoes consists of the following types of data:

a. Insecticide susceptibility (% mortality) and resistance mechanism
b. Species identification, insecticide susceptibility (% mortality), resistance intensity, and resistance mechanism
c. Insecticide susceptibility (% mortality), resistance intensity, and resistance mechanism
d. Species identification and insecticide susceptibility (% mortality)

7) Why did you choose the answer above? **Choose all that apply**.

a. Because a resistance profile must include all types of resistance data
b. Because, while resistance intensity is an important indicator of potential control failure, it is not considered part of the resistance profile
c. Because resistance intensity and mechanisms are not necessary to characterize a species' resistance profile
d. Because species identification allows you to assign resistance characteristics to a particular species rather than have potentially divergent data combined to a single value.

8) You have an insecticide that will last for 6 months if sprayed on the walls of houses. Based on the historical baseline data presented below (no interventions are present), what months would you perform spraying the following year?

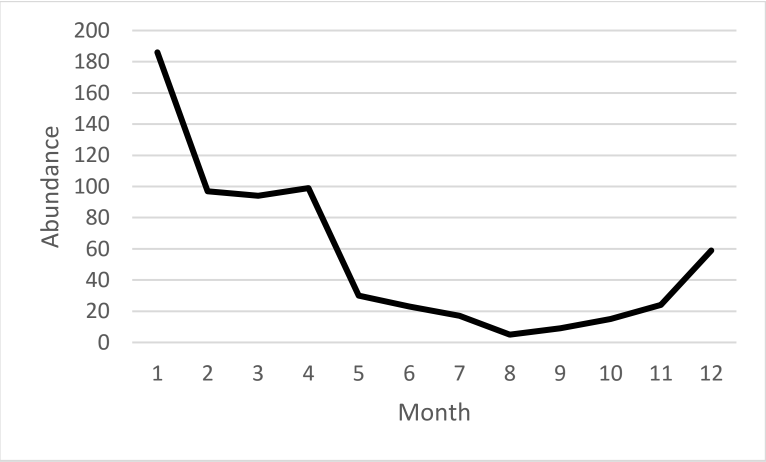

a. 8 or 9
b. 10 or 11
c. 12 or 1
d. 1 or 2

9) You have collected the following resistance data. Choose the best insecticide to use.

Insecticide class	% Mortality	Resistance mechanism	Resistance intensity
Pyrethroids	42	target site	1x
Carbamates	56	metabolic	5x
DDT	76	target site and metabolic	5x
Organophosphates	62	metabolic	5x

a. Pyrethroids
b. DDT
c. Carbamates

d. Organophosphates

10) The following graph presents data from an intervention monitoring tool.

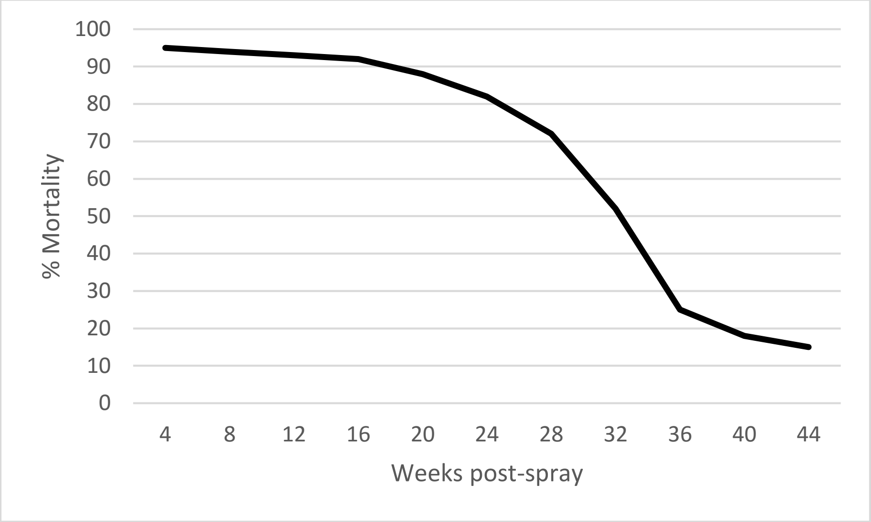

Choose the tool that was used to create this data.

a. HPLC
b. Cone assay
c. Field-based insecticide quantification assay
d. WHO tube assay

11) Two common data types that are monitored by vector control programmes are insecticide susceptibility and entomological inoculation rate (EIR).

For each statement, choose the type of data that it is most likely describing

A. Insecticide susceptibility
B. EIR

____This data will help to quantify the impact of an intervention on disease transmission

____This data will inform future insecticide choice

____This data will help to identify why an intervention is not having the desired impact

____This data will help to decide what time of year to start an indoor residual spray campaign

12) A vector control programme decides to use pyrethroid insecticides for their indoor residual spray campaign because they are cheap and effective, despite the presence of a very low level of pyrethroid resistance. They use pyrethroids for five consecutive years. Which of the following lines represent the most likely trend of resistance to pyrethroids during this time?

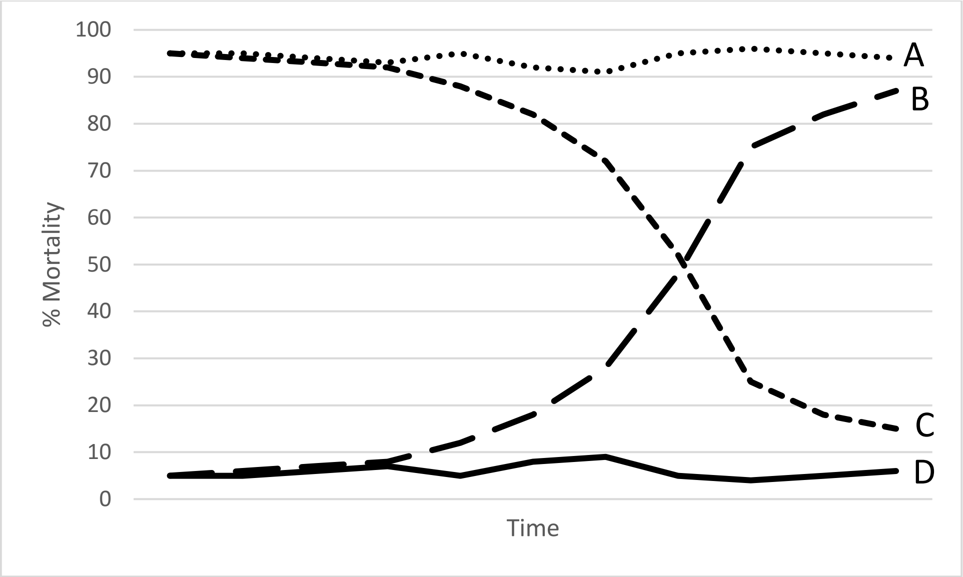

a. A
b. B
c. C
d. D

13) The programme stops using insecticides entirely for the subsequent five years. Which of the following lines represents the most likely trend of resistance to pyrethroids during this time?

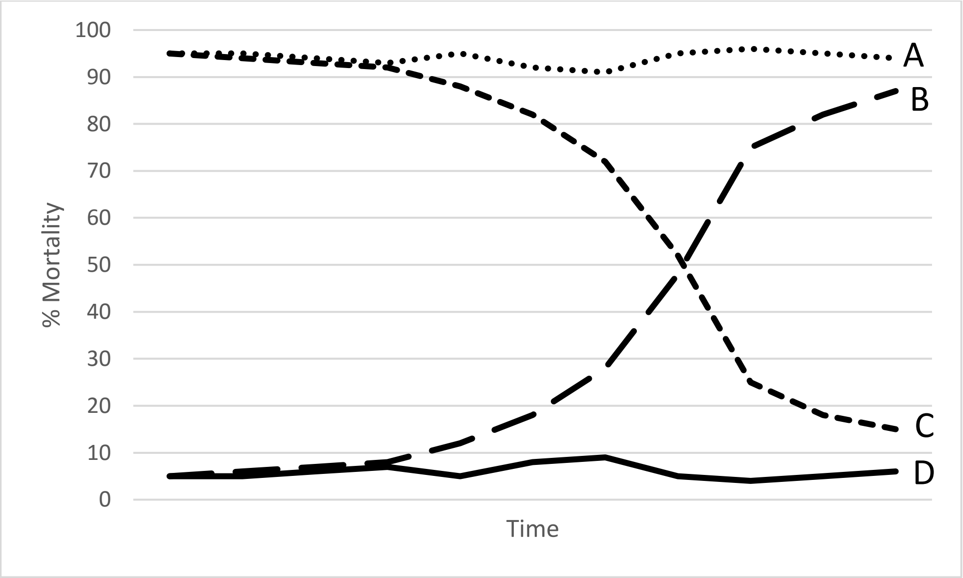

a. A
b. B
c. C
d. D

14) An indoor residual spray programme has determined that the quality and coverage of the intervention need to be improved. What is/are the likely action(s) that the programme completed to resolve both issues? Choose the best answer.

a. Collected more data on the amount of insecticide on the walls and the proportion of structures sprayed
b. Provided higher wages to the spray operators
c. Allowed more time for the spray operators to meet their targets
d. Retrained the spray operators and conducted community engagement activities

15) A vector control programme has collected susceptibility data for all insecticide classes and found that the mosquito population is resistant to everything. To maximize the useful life of the insecticides, they employ a common resistance management strategy. The following is a graph of the susceptibility data that they collect over the next five years. What strategy did they most likely employ?

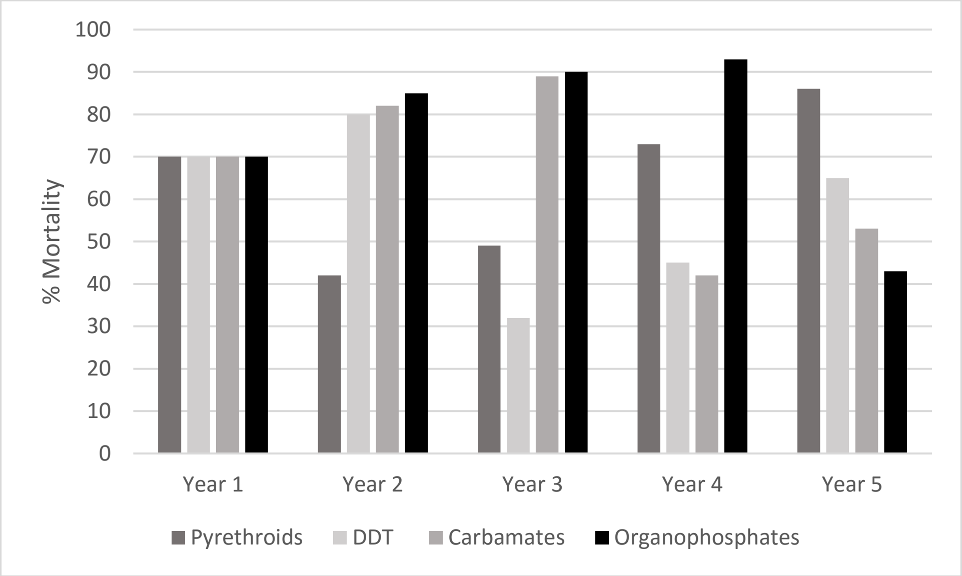

a. Insecticide rotations
b. Large scale mosaic
c. Small scale mosaic
d. Combination interventions (different insecticides used simultaneously in different interventions)

16) The four classes of insecticides currently available for public health use are:

a. Organochlorines (DDT), pyrethroids, organophosphates, carbamates
b. Organochlorines (DDT), pyrethroids, organophosphates, permethrin
c. Pyrethroids, organophosphates, carbamates, permethrin
d. Organochlorines (DDT), pyrethroids, carbamates, permethrin

17) Which of the following statements describes one way that target site resistance is different from metabolic resistance?

a. Target site resistance occurs outside the mosquito's body whereas metabolic resistance occurs inside

b. Target site resistance involves a change in physiology whereas metabolic resistance does not
c. Target site resistance involves a change in the binding site of the insecticide whereas metabolic resistance does not
d. Target site resistance is heritable whereas metabolic resistance is not

18) Which of the following is NOT a family of enzymes often involved in metabolic resistance?

a. ATP transferase
b. Esterase
c. Monooxygenase
d. Glutathione S transferase

19) How does a synergist work?

a. A synergist chemically changes the shape of the insecticide in the mosquito's body, making it more potent
b. A synergist blocks the activity of certain detoxifying enzymes, preventing them from detoxifying insecticides
c. A synergist increases binding of insecticides to their target site
d. A synergist prevents the excretory system of insects from working, making insecticides stay in the body longer

20) A mosquito population has metabolic resistance to pyrethroids conferred by monooxygenases. The susceptibility data looks like this:

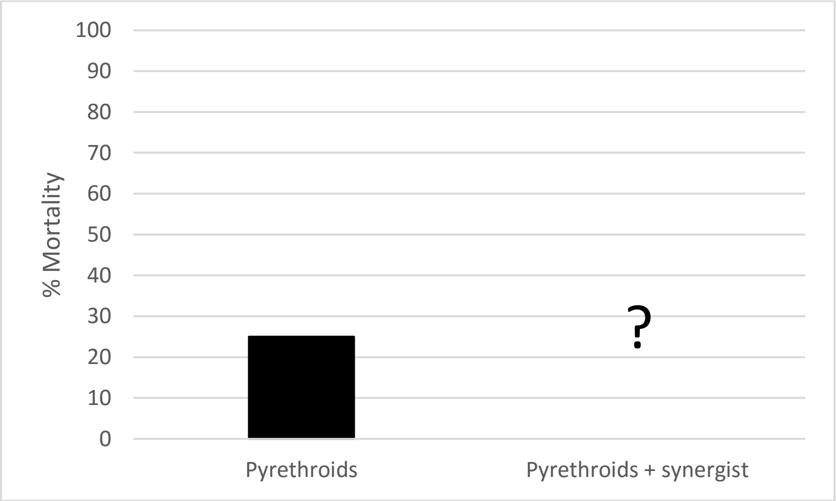

What will the susceptibility data look like if you also expose these mosquitoes to the synergist PBO?

a. Morality will stay the same
b. Mortality will be lower
c. Mortality will be higher
d. Mortality will drop to 0

21) Vectors that are resistant to pyrethroids and have kdr target-site resistance will probably

a. Be resistant to DDT

b. Be susceptible to DDT
c. Be resistant to all classes of insecticides
d. Be susceptible to all classes of insecticides

22) Why did you choose the answer above?

a. Because DDT has the same mode of action as pyrethroids
b. Because DDT has a different mode of action as pyrethroids
c. Because kdr is a universal resistance mechanism
d. Because kdr only interacts with synergists
