# Supplementary material for: Evaluation of a game-based training course to build capacity for insecticide resistance management in vector control programmes
Source: PLoS One. 2020 Oct 15;15(10):e0240514. doi: 10.1371/journal.pone.0240514 (PMC7561117; doi:10.1371/journal.pone.0240514)
Supplement: S2 Appendix — (PDF) [file pone.0240514.s002.pdf]

ResistanceSim - Self-efficacy questionnaire (Before)

Indicate below how certain you are that you can do these things by choosing a number from 0 to 10.

0 indicates that you cannot do the activity. 10 indicates that you can do the activity.

Values in between indicate that you can sometimes do the activity.

1. Participant Number

____

Identifying important data

2. I am confident that I can identify the data that is needed to make an informed vector control intervention choice.

Mark only one oval.

	1	2	3	4	5	6	7	8	9	10	
I cannot do this	◯	◯	◯	◯	◯	◯	◯	◯	◯	◯	I can do this

3. I am confident that I can identify the data that is needed to monitor the impact of a vector control intervention.

Mark only one oval.

	1	2	3	4	5	6	7	8	9	10	
I cannot do this	◯	◯	◯	◯	◯	◯	◯	◯	◯	◯	I can do this

4. I am confident that I can identify the data that is needed to determine the quality of a vector control intervention.

Mark only one oval.

	1	2	3	4	5	6	7	8	9	10	
I cannot do this	◯	◯	◯	◯	◯	◯	◯	◯	◯	◯	I can do this

Choosing methods to get that data

5. I am confident that I can choose an appropriate mosquito collection method to get the entomological data I want.

Mark only one oval.

	1	2	3	4	5	6	7	8	9	10	
I cannot do this	◯	◯	◯	◯	◯	◯	◯	◯	◯	◯	I can do this

6. I am confident that I can choose an appropriate bioassay or laboratory assay to get the insecticide resistance data I want.

Mark only one oval.

	1	2	3	4	5	6	7	8	9	10	
I cannot do this	◯	◯	◯	◯	◯	◯	◯	◯	◯	◯	I can do this

7. I am confident that I can choose an appropriate method to determine the quality of an intervention.

Mark only one oval.

	1	2	3	4	5	6	7	8	9	10	
I cannot do this	◯	◯	◯	◯	◯	◯	◯	◯	◯	◯	I can do this

Interpreting data

8. I am confident that I can correctly interpret entomological and resistance data to make the best decision regarding whether to use a particular vector control tool.

Mark only one oval.

	1	2	3	4	5	6	7	8	9	10	
I cannot do this	◯	◯	◯	◯	◯	◯	◯	◯	◯	◯	I can do this

Operational decision-making

9. I am confident that I can plan an appropriate monitoring and evaluation strategy for a vector control program.

Mark only one oval.

	1	2	3	4	5	6	7	8	9	10	
I cannot do this	◯	◯	◯	◯	◯	◯	◯	◯	◯	◯	I can do this

10. I am confident that I can prioritize vector control and monitoring activities given budgetary and time constraints.

Mark only one oval.

	1	2	3	4	5	6	7	8	9	10	
I cannot do this	◯	◯	◯	◯	◯	◯	◯	◯	◯	◯	I can do this

11. I am confident that I can identify appropriate strategies to ensure that vector control interventions are sustainable.

Mark only one oval.

	1	2	3	4	5	6	7	8	9	10	
I cannot do this	◯	◯	◯	◯	◯	◯	◯	◯	◯	◯	I can do this

12. I am confident that I can "troubleshoot" a vector control intervention that is not having the expected impact.

Mark only one oval.

	1	2	3	4	5	6	7	8	9	10	
I cannot do this	◯	◯	◯	◯	◯	◯	◯	◯	◯	◯	I can do this

Additional activities

Please describe any other activities associated with insecticide resistance management that were not included in this survey, and indicate your confidence in you ability to complete these activities

13. Activity____

Mark only one oval.

	1	2	3	4	5	6	7	8	9	10	
I cannot do this	◯	◯	◯	◯	◯	◯	◯	◯	◯	◯	I can do this

14. Activity____

Mark only one oval.

	1	2	3	4	5	6	7	8	9	10	
I cannot do this	◯	◯	◯	◯	◯	◯	◯	◯	◯	◯	I can do this

15. Activity____

____

____

Mark only one oval.

	1	2	3	4	5	6	7	8	9	10	
I cannot do this	◯	◯	◯	◯	◯	◯	◯	◯	◯	◯	I can do this

16. Additional Comments

____

____

____

____

____

This content is neither created nor endorsed by Google.

Google Forms
