# Supplementary material for: Evaluation of a game-based training course to build capacity for insecticide resistance management in vector control programmes
Source: PLoS One. 2020 Oct 15;15(10):e0240514. doi: 10.1371/journal.pone.0240514 (PMC7561117; doi:10.1371/journal.pone.0240514)
Supplement: Attachment — Submitted filename: Comments PONE-D-20-12756.pdf [file pone.0240514.s007.pdf]

Reviewers' comments

PONE-D-20-12756: **Evaluation of a game-based training course to build capacity for insecticide resistance management in vector control programmes**

**Section**	**Comments**
**Abstract**	• Authors should consider expanding on how the training was evaluated i.e. through a pre and post-test and interviews so that the abstract clearly outlines the study done
**Materials and methods**	• I strongly feel that the authors study design should have included a cohort where the usual IRM training was done through lectures and practical (usual approach used). This would have allowed comparison between these two approaches and prove beyond doubt that their approach is more effective.<br>• I am sceptical on the approach of doing group training and evaluate outcomes at individual level.<br>• Although participant selection was well described it is not clear what informed selection of Ethiopia and Zambia out of all sub-Saharan countries. There should be some form of criteria why these countries were chosen considering the diversity of sub-Saharan Africa in malaria transmission, advances in IRM, technology etc. these factors confound the outcome of the study.

	• Data analysis: Authors should consider first analysing country data separately before combining the data. The dynamics and understanding of IR in these two settings are different and combining data without exploring these differences is an oversight that has implication on the conclusions reached.
**Results**	• Raw data on results presented in lines 223 -229 should be presented in the main document and or supplementary. This data is critical as it has implication on the outcomes of this study.<br>• Lines 244 – 247 "To investigate the relationship between knowledge acquisition and the level of pre course knowledge, the participants were divided into four quartiles, according to their results in the knowledge test before the training. There were eleven participants per quartile, Q1 represents the lowest scores and Q4 the highest scores." Did the authors divide the participants into four equal quarters then worked score divisions according to these groups? It is highly unlikely to have equal participants in each quarter if the division was solely based on scores as reported here.<br>• Qualitative Thematic analysis: It was better to categorise outcomes so that reporting becomes quantitative. It is subjective to have statements such as line 304 "…. most participants used a

	range of positive affective terms in relation to both….”; line 313 “Their game experience with ResistanceSim and Resistance101 was distinctly different” Line 317 “Numerous participants mentioned the animations of Resistance101.”; Line 337 “Quite a few participants mentioned feedback as a positive feature of the game.” etc.
**Conclusion**	• Authors highlighted sample size as their limitation therefore their conclusion should be addressed in the context of these two countries not generalisation<br>• One of the recommendation should have been addressing language challenges in future through the use of translators. Authors clearly indicated and rightly so that language barrier could have impacted the interviews, and thus the thematic analysis, and possibly other aspects of the training.<br>• Another recommendation is doing the study in a stratified manner to cover the different sub-Saharan geographical, transmission setting, technological and educational advancement differences etc
