# Supplementary material for: Evaluation of a game-based training course to build capacity for insecticide resistance management in vector control programmes
Source: PLoS One. 2020 Oct 15;15(10):e0240514. doi: 10.1371/journal.pone.0240514 (PMC7561117; doi:10.1371/journal.pone.0240514)
Supplement: Attachment — Submitted filename: Response to reviewers.docx [file pone.0240514.s008.docx]

04 September 2020

Letter to accompany the revision of the manuscript titled “Evaluation of a game-based training course to build capacity for insecticide resistance management in vector control programmes” by Dormann and colleagues.

Dear Dr. Brooke,

We would like to thank you, reviewer 1, and Dr. Munhenga for taking the time to provide valuable comments to improve this manuscript. We have considered each of the points raised by Dr Munhenga and our responses are indicated below.

1) *Authors should consider expanding on how the training was evaluated i.e. through a pre and post-test and interviews so that the abstract clearly outlines the study done*

Thank you for this suggestion, we have added this information on lines 26-27.

2) *I strongly feel that the authors study design should have included a cohort where the usual IRM training was done through lectures and practical (usual approach used). This would have allowed comparison between these two approaches and prove beyond doubt that their approach is more effective.*

We agree with the reviewer that this would have allowed us to conclude with greater certainty that the game-based method was a better approach to training. The co-authors in fact had many discussions about this during the study design phase. In the end, we decided on the pre-post approach primarily because there is currently no “gold standard” training approach or curriculum for IRM that we are aware of, other than the WHO handbook (GPIRM). While lectures and practicals are typical approaches used in other types of training, we did not feel this justified the assumption that this would be a valid control group for our IRM training. Recognising this limitation, we decided instead to increase the weight of the qualitative analysis generated from participant interviews to try to explore the potential benefits of the game-based approach. In our discussion, we have been purposeful with the use of our language to make sure that we do not overstate the impact of our results.

3) *I am sceptical on the approach of doing group training and evaluate outcomes at individual level.*

Thank you for expressing this concern. Group training was the most feasible way to train many individuals and is likely the method that will be used if the game-based training course was rolled out to a wider audience. The impact of the training (on knowledge, self-efficacy, and perceptions) was captured through individual assessments, but results were analysed and presented at group level. The alternative, that metrics from assessment materials were combined into a pre-course value and a post-course value, would have prevented any meaningful comparison of the data with statistical tests since it would not be possible to calculate means. In addition, we would have lost the ability to compare results of subsets of participants based on certain characteristics (ie pre-course performance).

4) *Although participant selection was well described it is not clear what informed selection of Ethiopia and Zambia out of all sub-Saharan countries. There should be some form of criteria why these countries were chosen considering the diversity of sub-Saharan Africa in malaria transmission, advances in IRM, technology etc. these factors confound the outcome of the study.*

A statement about the criteria for selecting these countries has been added to the manuscript on lines 77-79. To provide a bit more context, these 2 countries were partner countries in a larger consortium that also involved Cameroon and Cote D’Ivoire. Ethiopia and Zambia were better equipped to organise the training in terms of stakeholder engagement and logistics. Cameroon and Cote D’Ivoire recently participated in separate trainings, but we felt that the time between trainings in Zambia/Ethiopia and Cameroon/Cote D’Ivoire prevented us from meaningfully analysing the data as a single study. We do not believe this compromises the outcomes of the study presented in this manuscript

5) *Data analysis: Authors should consider first analysing country data separately before combining the data. The dynamics and understanding of IR in these two settings are different and combining data without exploring these differences is an oversight that has implication on the conclusions reached.*

We did analyse country data separately before deciding to present it in the way that we have. The country-level analysis revealed that in Ethiopia, pre-course knowledge and self-efficacy scores were higher, and change (post-course) in both scores was lower than in Zambia. As the reviewer points out, this is likely because Ethiopian participants were more versed in IRM than the Zambian participants. However, we believe the important result is not in the difference between the two countries, but in the difference in improvement between those who start with higher levels of knowledge/self-efficacy vs those with lower levels. This is why we decided to present results by pre-course performance, and we believe this makes the results more applicable to different contexts. We have left the results unchanged, but we have included the country affiliations in the raw data (new S3 Appendix) in case readers would like to explore this in more detail.

6) *Raw data on results presented in lines 223 -229 should be presented in the main document and or supplementary. This data is critical as it has implication on the outcomes of this study.*

We agree, this has now been added as S3 appendix (and subsequent appendices renamed).

7) *Lines 244 – 247 “To investigate the relationship between knowledge acquisition and the level of pre course knowledge, the participants were divided into four quartiles, according to their results in the knowledge test before the training. There were eleven participants per quartile, Q1 represents the lowest scores and Q4 the highest scores.” Did the authors divide the participants into four equal quarters then worked score divisions according to these groups? It is highly unlikely to have equal participants in each quarter if the division was solely based on scores as reported here.*

Yes, you are correct. We ranked the participants according to pre-course knowledge score, and then divided the ranked list into 4 groups of equal participants. The wording of this section has been changed to improve clarity.

8) *Qualitative Thematic analysis: It was better to categorise outcomes so that reporting becomes quantitative. It is subjective to have statements such as line 304 “…. most participants used a range of positive affective terms in relation to both….”; line 313 “Their game experience with ResistanceSim and Resistance101 was distinctly different” Line 317 “Numerous participants mentioned the animations of Resistance101.”; Line 337 “Quite a few participants mentioned feedback as a positive feature of the game.” etc*.

We understand the concern expressed here. However, given that this section is presenting qualitative results from participant interviews, the goal is not to quantify how many participants felt one way or another, but rather to explore the common themes that were mentioned in participant interviews. As such, we have revised the text in many circumstances (those mentioned above as well as others) to omit subjective references to participant numbers or other quantitative measures. In some cases, we felt that it was important to make a distinction that some, but not all, participants felt a certain way. In these cases we have left the language as written.

9) *Authors highlighted sample size as their limitation therefore their conclusion should be addressed in the context of these two countries not generalisation*

Text has been added to the discussion and conclusions sections to highlight that the conclusions apply to the specific participant/country contexts.

10) *One of the recommendation should have been addressing language challenges in future through the use of translators. Authors clearly indicated and rightly so that language barrier could have impacted the interviews, and thus the thematic analysis, and possibly other aspects of the training.*

This has been added in the limitations section.

11) *Another recommendation is doing the study in a stratified manner to cover the different sub-Saharan geographical, transmission setting, technological and educational advancement differences etc*

This was previously included in the limitations section but has now been expanded.

Sincerely,

Edward Thomsen, PhD

Department of Vector Biology
